# Seasonal Influence on Rumen Microbiota, Rumen Fermentation, and Enteric Methane Emissions of Holstein and Jersey Steers under the Same Total Mixed Ration

**DOI:** 10.3390/ani11041184

**Published:** 2021-04-20

**Authors:** Mahfuzul Islam, Seon-Ho Kim, A-Rang Son, Sonny C. Ramos, Chang-Dae Jeong, Zhongtang Yu, Seung Ha Kang, Yong-Il Cho, Sung-Sill Lee, Kwang-Keun Cho, Sang-Suk Lee

**Affiliations:** 1 Ruminant Nutrition and Anaerobe Laboratory, Department of Animal Science and Technology, Sunchon National University, Suncheon 57922, Korea; mislam.mipa@sau.edu.bd (M.I.); mhs0425@hanmail.net (S.-H.K.); sonarang7@naver.com (A-R.S.); ynnosomarc@yahoo.com.ph (S.C.R.); cdvf12@hanmail.net (C.-D.J.); 2 Department of Microbiology and Parasitology, Sher-e-Bangla Agricultural University, Dhaka 1207, Bangladesh; 3 Department of Animal Sciences, The Ohio State University, Columbus, OH 43210, USA; yu.226@osu.edu; 4 Faculty of Medicine, Diamantina Institute, The University of Queensland, Brisbane, QLD 4072, Australia; kansbio@gmail.com; 5 Animal Disease and Diagnostic Laboratory, Department of Animal Science and Technology, Sunchon National University, Suncheon 57922, Korea; ycho@scnu.ac.kr; 6 Institute of Agriculture and Life Science and University-Centered Labs, Gyeongsang National University, Jinju 52828, Korea; lss@gnu.ac.kr; 7 Department of Animal Resources Technology, Gyeongnam National University of Science and Technology, Jinju 52725, Korea; chotwo2@gntech.ac.kr

**Keywords:** enteric methane emissions, seasonal changes, rumen microbiota, steers, volatile fatty acids

## Abstract

**Simple Summary:**

The rumen microbiome plays a significant role in the breakdown of dietary substrates in the rumen and thus provides essential nutrients to the animals. However, methane (CH_4_) production by methanogens drains dietary energy. Therefore, manipulation of the rumen microbiome is one way to improve animal performance and reduce enteric methane emissions from ruminants. However, most previous studies have focused on dairy cattle at specific time points; thus, little is known about the rumen microbiome of steers and seasonal effects. This study aimed to compare the rumen microbiome, rumen fermentation and enteric CH_4_ emissions of Holstein and Jersey steers over different seasons. Both season and breed affected the rumen microbiome and rumen fermentation, while only breed affected enteric CH_4_ emissions. Our results suggest that both season and breed must be considered when manipulating the rumen microbiome to enhance animal performance. In addition, breed should be taken into consideration to reduce CH_4_ emissions from steers.

**Abstract:**

Seasonal effects on rumen microbiome and enteric methane (CH_4_) emissions are poorly documented. In this study, 6 Holstein and 6 Jersey steers were fed the same total mixed ration diet during winter, spring, and summer seasons under a 2 × 3 factorial arrangement for 30 days per season. The dry matter intake (DMI), rumen fermentation characteristics, enteric CH_4_ emissions and rumen microbiota were analyzed. Holstein had higher total DMI than Jersey steers regardless of season. However, Holstein steers had the lowest metabolic DMI during summer, while Jersey steers had the lowest total DMI during winter. Jersey steers had higher CH_4_ yields and intensities than Holstein steers regardless of season. The pH was decreased, while ammonia nitrogen concentration was increased in summer regardless of breed. Total volatile fatty acids concentration and propionate proportions were the highest in winter, while acetate and butyrate proportion were the highest in spring and in summer, respectively, regardless of breed. Moreover, Holstein steers produced a higher proportion of propionate, while Jersey steers produced a higher proportion of butyrate regardless of season. Metataxonomic analysis of rumen microbiota showed that operational taxonomic units and Chao 1 estimates were lower and highly unstable during summer, while winter had the lowest Shannon diversity. Beta diversity analysis suggested that the overall rumen microbiota was shifted according to seasonal changes in both breeds. In winter, the rumen microbiota was dominated by *Carnobacterium jeotgali* and *Ruminococcus bromii*, while in summer, *Paludibacter propionicigenes* was predominant. In Jersey steers, *Capnocytophaga cynodegmi*, *Barnesiella viscericola* and *Flintibacter butyricus* were predominant, whereas in Holstein steers, *Succinivibrio dextrinosolvens* and *Gilliamella bombicola* were predominant. Overall results suggest that seasonal changes alter rumen microbiota and fermentation characteristics of both breeds; however, CH_4_ emissions from steers were significantly influenced by breeds, not by seasons.

## 1. Introduction

Global warming, caused by the increasing production of greenhouse gases from different sources including agriculture and livestock, is of great global concern [1,2]. Therefore, it is a prerequisite to increase the number of heat tolerant breeds globally. This is because sustainable animal production depends on environmental temperature, and thermoneutrality is needed for normal metabolism and physiological activities. Both seasonal stressors, either cold or heat, can negatively affect animal performance [3,4,5,6,7,8,9]. During cold stress, increased maintenance energy is required to retain body temperature, and feed efficiency is greatly hampered [3]. In contrast, it is not uncommon for cattle to reduce dry matter intake (DMI) and rumen motility during heat stress [10]. Jersey cows had better adaptation capabilities to heat stress compared to Holstein cows [11,12]; however, Holstein cows are well adapted to lower temperatures (the lower and upper critical temperature varies from −15 °C to 22 °C) [13]. It is well known that Holstein and Jersey are two important dairy breeds, and the contribution steers of these breeds make to beef production is of considerable value [14,15]. However, most previous studies have focused on dairy breeds and little is known about the steers of dairy breeds.

Members of the rumen microbiota, including bacteria, protozoa, fungi, and archaea, can ferment a wide variety of ingested feedstuffs to subsequently produce volatile fatty acids (VFAs), such as acetate, propionate, and butyrate, which are then absorbed by the cattle for energy metabolism and protein synthesis [16,17,18,19]. Simultaneously, carbon dioxide (CO_2_), hydrogen (H_2_) and formic acid are produced as end products. Enteric methane (CH_4_) can also be produced by methanogens through methanogenesis [20,21,22]. CH_4_ is an indicator of dietary gross energy losses, and it has a negative environmental impact contributing to global warming [23,24]. Previous studies revealed that several factors, including diet, feed additives, host genetics, age, and physiological state affect the rumen microbiomes, rumen fermentation characteristics, and CH_4_ production [25,26,27,28,29,30,31,32,33,34]. O’Hara et al. [28] reported an association between the rumen microbiome and its fermentation products with feed efficiency and CH_4_ emissions. They also reported that Firmicutes, Bacteroidetes, and Proteobacteria were the dominant bacterial phyla that can ferment a wide variety of dietary carbohydrates and peptides. However, few studies have focused on the seasonal influence on the rumen microbiome and CH_4_ emissions. Li et al. [35] conducted an experiment examining seasonal effects on microbial diversity in the feces of Holstein dairy cows and stated that fecal microbial diversity and composition varied at different temperature humidity index (THI) values. Noel et al. [36] reported a shift in the digesta-adherent rumen microbiome of Holstein dairy cow grazing pastures over the seasons. However, the fecal microbiome in the former study and the pasture grazing cattle of the other study did not completely represent the rumen microbiome changes in feedlot cattle over the seasons. Moreover, the majority of previous studies have focused on dairy cattle, whereas steers are much less researched. To the best of our knowledge, no previous study has examined the influence of seasonal stress on the rumen microbiomes, its fermentation parameters, and enteric CH_4_ emissions of Holstein and Jersey steers fed the same total mixed ration (TMR). We hypothesized that season and breed can influence the rumen microbiota, rumen fermentation, and enteric CH_4_ emissions. In this context, the present study was conducted (i) to evaluate whether the rumen fermentation characteristics and enteric methane emissions of Holstein and Jersey steers fed the same TMR over different seasons are similar and (ii) to determine to what extent the diversity and composition of the rumen microbiome vary between breeds and among seasons.

## 2. Materials and Methods

### 2.1. Animals, Experimental Design, and Diet

Animal experiments were conducted at the Sunchon National University (SCNU) animal farm. Laboratory analyses were performed at the Ruminant Nutrition and Anaerobe Laboratory, Department of Animal Science and Technology, SCNU, Jeonnam, Korea. This study was conducted during the period from December 2018 to August 2019 and consisted of three seasons, winter (mid-December to mid-January), spring (mid-March to mid-April), and summer (mid-July to mid-August).

Six Holstein (bodyweight: 508.92 ± 7.95 kg; age: 17.33 ± 0.52 months) and six Jersey (bodyweight: 392.75 ± 30.85 kg; age: 17.67 ± 1.03 months) steers, both non-cannulated, were fed the same TMR diet (Table 1) during winter, spring, and summer seasons under a 2 × 3 factorial arrangement (2 breeds and 3 seasons as factors) for 30 days per season. Each of the 30-day seasons was divided into an initial 25 days of diet adaptation and 5 days of data collection, with the first three days for enteric CH_4_ emissions and the fifth day for rumen fluid sampling. All steers were kept in individual stalls with feeding and water facilities. The steers were offered the TMR once a day at 09:00 a.m. with a 5–10% diet refusal. Feed intake (FI) was measured as the difference between the feed offered and refusal. DMI was calculated from FI based on the dry matter content of TMR. The TMR was sampled twice (at days 7 and 21) during the feeding trial, and the dry matter content was determined using a hot-air oven at 65 °C for 72 h. The chemical composition of the TMR was analyzed following standard methods [37]. The contents of neutral detergent fiber (NDF) and acid detergent fiber (ADF) were determined according to the protocols described by Van Soest et al. [38] and Van Soest [39], respectively.

### 2.2. Recording of THI

The ambient temperature (°C) and relative humidity (%) of the experimental shed were recorded for the last 10 days of the seasonal experimental periods using the Testo 174H Mini data logger (West Chester, PA, USA). The THI was calculated as THI = (0.8 × maximum ambient temperature) + [% relative humidity/100 × (mean ambient temperature − 14.4)] + 46.4 [40].

### 2.3. Enteric CH_4_ Measurements

Enteric CH_4_ emissions were measured using a GreenFeed (GF) unit, also called automated head chamber system, (C-Lock Inc., Rapid City, SD, USA), as described by Hammond et al. [41] and Hristov et al. [42], with minor modifications. Briefly, all steers were allowed to adapt to the GF unit before the experiment started in each season to mitigate any associated psychological stress. CH_4_ emissions were measured for each steer at eight different time points (00:00, 03:00, 06:00, 09:00, 12:00, 15:00, 18:00, and 21:00) for three consecutive days during each seasonal measurement period. The GF unit was installed in one corner of a large pen. At each measurement time, all steers were successively moved from their stalls to this pen. Each steer was allowed to access the GF unit for approximately 10 min. Molasses-coated concentrated pellets (250–300 g/visit) were used to attract the animals to the GF unit and to ensure a proper head-down position within the hood for the duration of the measurement. The amount of the pellets ingested by each steer per day was not included in the DMI calculation. The entry and exit times for each animal, standard gas calibration, and CO_2_ recovery data were recorded and sent to C-Lock Inc. The calculated data were received via a web-based data management system, and CH_4_ emissions were calculated as CH_4_ production (g/d), CH_4_ yield (g/kg DMI), and CH_4_ intensity (g/kg BW^0.75^).

### 2.4. Sample Collection and Processing

In each season, rumen fluid samples were collected using stomach tubing from each of the steers at two different time points: before feeding (0 h) and 6 h after feeding on the last day of the experiment. To minimize contamination from saliva, the first 300 mL of rumen fluid samples were discarded. The pH was immediately measured using a pH meter (Seven CompactTM pH/Ion meter S220, Mettler Toledo, Switzerland) after collection. At the same time, three separate aliquots were made from the rumen fluid samples collected from each steer, transported to the laboratory using dry ice, and stored at −80 °C until subsequent analysis of ammonia nitrogen (NH_3_-N), volatile fatty acid (VFA), and rumen microbiota was performed.

### 2.5. NH_3_-N and VFA Analyses

The concentration of NH_3_-N was measured colorimetrically using a Libra S22 spectrophotometer (CB40FJ; Biochrom Ltd., Cambourne, UK) following the protocol described by Chaney and Marbach [43]. VFA concentration was measured according to the methods described by Han et al. [44] and Tabaru et al. [45] using high-performance liquid chromatography (HPLC; Agilent Technologies 1200 series, Waldbronn, Germany). A UV detector (set at 210 and 220 nm), a METACARB87H column (Varian, Palo Alto, CA, USA), and a buffered solvent (0.0085 N H_2_SO_4_) at a flow rate of 0.6 mL/min were used to perform HPLC.

### 2.6. DNA Extraction and Metataxonomic Analysis

Rumen fluid samples (two of each of the 12 steers (*n* = 24) over three seasons; 72 in total) were sent to Macrogen Inc. (Seoul, Korea) for DNA extraction and metataxonomic analysis of the rumen microbiota. Briefly, DNA was extracted using a PowerSoil^®^ DNA Isolation Kit (Cat. No. 12888, MO BIO) following the manufacturer’s protocol [46]. The quality and quantity of DNA were assessed using PicoGreen and Nanodrop. Illumina 16S Metagenomic Sequencing Library protocols were used to prepare the amplicon library of each sample, using two-step PCR amplification of the V3-V4 region of the 16S rRNA genes with the primers Bakt_341F (5-AGATGTGTATAAGAGACAG-3) and Bakt_805R (5-GATGTGTATAAGAGACAGG-3) [47] (25 cycles in the first PCR), with multiplexing indices and Illumina sequencing adapters introduced in the second PCR (10 cycles). Products of the first and second PCR were purified using Ampure beads (Agencourt Bioscience, Beverly, MA, USA). Individual amplicon libraries were normalized after quantification using PicoGreen, size-verified using a TapeStation DNA ScreenTape D1000 (Agilent Technologies), pooled at an equimolar ratio, and then sequenced on a MiSeq system (Illumina, San Diego, CA, USA) using the 2 × 300 bp kit. Raw sequence data were trimmed using Trimmomatic (v0.38) [48], and paired reads were merged using the FLASH (1.2.11) software [49]. Sequences shorter than 400 bp were discarded. rDnaTools (https://github.com/PacificBiosciences/rDnaTools) was used to identify and remove chimeric sequences. Samples were subsampled to an even depth of 10,000 sequences per sample to avoid bias generated at different sequencing depths. The filtered sequences were clustered into operational taxonomic units (OTUs) at 97% sequence similarity using CD-HIT-OTU [50]. The representative sequence of each OTU was compared against the 16S Microbial DB of NCBI for taxonomic assignment (https://www.ncbi.nlm.nih.gov/refseq/targetedloci/16S_process/, accessed on 19 June 2020) using BLASTN (v2.9.0+) [51]. Alpha diversity measurements including the Shannon diversity index and Chao1 richness estimate were determined using QIIME (v1.8). Box plots (for observed OTUs, Chao 1, Shannon index) and Venn diagrams depicting OTU overlapping (core rumen microbiome) were constructed using the Metagenomics core microbiome exploration tool (MetaCoMET; https://probes.pw.usda.gov/MetaCoMET/MetaCoMET_start.php, accessed on 9 November 2020). Principal Coordinate Analyaia (PCoA) was performed based on the Bray-Curtis distance dissimilarity matrix using the phyloseq package of Microbiome Analyst (https://www.microbiomeanalyst.ca/, accessed on 4 March 2021) with the normalized data to assess differences in overall rumen microbiota among seasons of both breeds.

### 2.7. Statistical Analysis

All data on DMI, CH_4_ emissions, and rumen fermentation were analyzed using the Mixed procedure of SAS (version 9.4; SAS Institute Inc., Cary, NC, USA) [52]. The model included the fixed effects season, breed, and an interaction term of season and breed, and the random effects included individuals nested within breeds. The relative abundance of individual taxa of the rumen microbiota was analyzed using a Kruskal–Wallis test with compositional normalized data. Average values of the different time points were used for the analysis of DMI, CH_4_ emissions, rumen fermentation, and the rumen microbiota. Additional analysis of seasonal variation within an individual breed as well as breed variation in each season were performed by GLM along with Duncan’s Multiple Range Test. Statistical significance was declared at *p* < 0.05.

## 3. Results

### 3.1. THI of the Experimental Period

The recorded ambient temperature, relative humidity, and THI of the three different seasons (winter, spring, and summer) are presented in Table 2. Based on THI, the entire experimental period was designated as three stress categories, namely, cold stress, no stress, and heat stress for the winter, spring, and summer season, respectively.

### 3.2. DMI and Enteric CH_4_ Emissions

The DMI and CH_4_ emissions of Holstein and Jersey steers fed the same TMR diet varied in a season-dependent manner (Table 3). Total DMI was highest in the spring followed by summer and winter; however, the highest and the lowest metabolic DMI was observed in the spring and the summer, respectively, compared to winter season irrespective of breed (*p* < 0.01 for both). Furthermore, total DMI was higher in Holstein than Jersey steers regardless of season (*p* < 0.01). However, the seasonal trend of an individual breed showed that Holstein steers had the highest total DMI in spring and the lowest metabolic DMI in summer, while Jersey steers had the lowest total DMI in the winter season (*p* < 0.05 for both). Compared to Jersey steers, the metabolic DMI trends of Holstein steers were higher during the spring and lower during the summer season (*p* < 0.05 for both). CH_4_ production, yield, and intensity were not affected either by season or the interaction between season and breed (*p* > 0.05). However, the CH_4_ yield, and intensity were higher in the Jersey than the Holstein steers regardless of season (*p* < 0.01 for both).

### 3.3. Rumen Fermentation Characteristics

Seasonal variation in the rumen fermentation parameters of Holstein and Jersey steers were evaluated, and the results are presented in Table 4. The lowest pH and the highest NH_3_-N concentration were observed in the summer compared to other seasons regardless of breed (*p* = 0.02 for pH and *p* < 0.01 for NH_3_-N). Furthermore, pH was higher in Holstein steers, while NH_3_-N was higher in Jersey steers regardless of season (*p* = 0.03 for both). Total VFA was higher in winter, while lower in summer compared to spring season irrespective of breed (*p* < 0.01). Acetate proportion was higher in spring, while propionate proportion was higher in winter, and butyrate proportion was higher in summer compared to other seasons regardless of breed (*p* < 0.01 for all). Furthermore, propionate proportion was higher in Holstein, while butyrate was higher in Jersey steers regardless of season (*p* < 0.01 for both). The A:P ratio was lower in winter compared to other seasons regardless of breed (*p* < 0.01). Moreover, Holstein had a lower A:P ratio than Jersey steers regardless of season (*p* < 0.01).

### 3.4. Species Richness, Diversity, and Composition of the Rumen Microbiota

**Figure 1:**
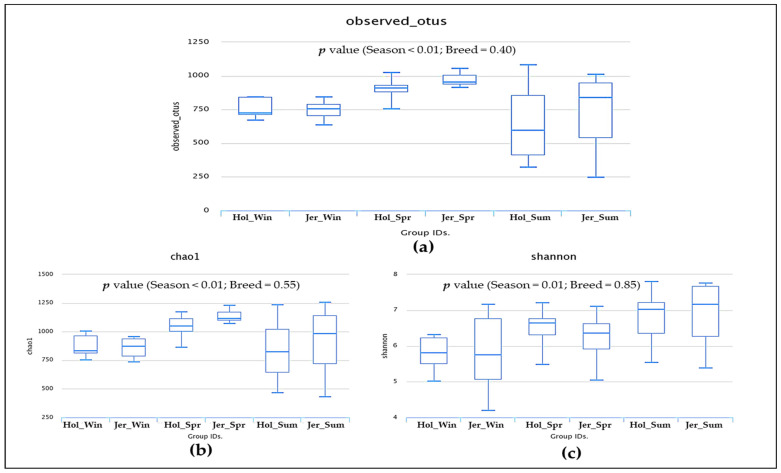
Box plots of observed OTUs (**a**), Chao1 estimates (**b**), and Shannon diversity index (**c**) of Holstein and Jersey steers at different seasons. Hol, Holstein steer; Jer, Jersey steer; Win, winter; Spr, spring; Sum, summer.

**Figure 2:**
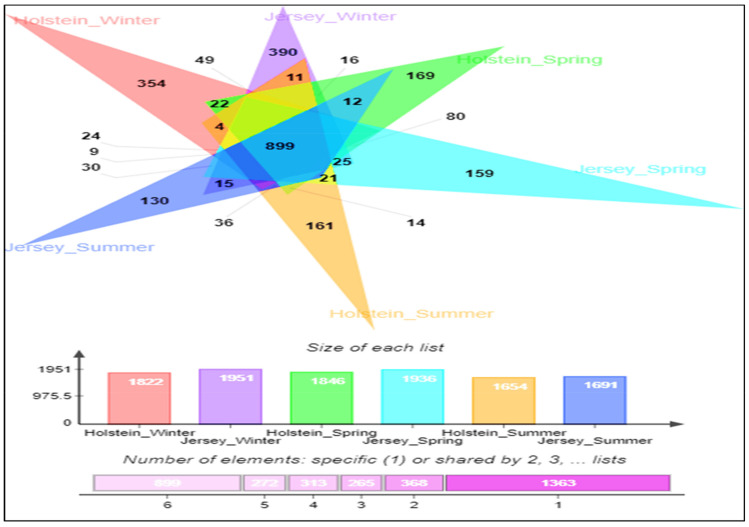
Venn diagram OTU overlapping (core rumen microbiome) of Holstein and Jersey steers at different seasons.

**Figure 3:**
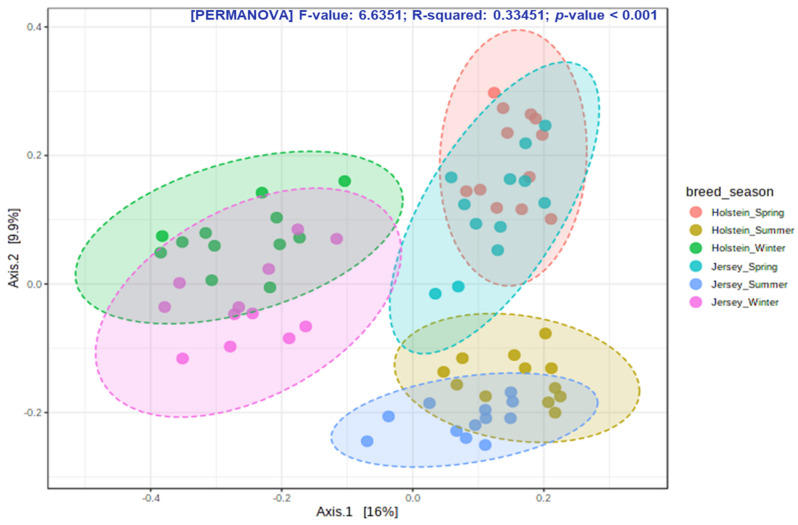
Principal coordinate analysis (PCoA) plot based on Bray-Curtis dissimilarity matrix showing the seasonal shifting of the overall rumen microbiota of Holstein and Jersey steers.

A total of 2,960,444 quality-filtered sequence reads were retained from 10,734,271 raw reads produced from 72 rumen fluid samples. The highest average OTU numbers and Chao 1 richness estimate were recorded in spring compared to other seasons regardless of breed; however, high variation of richness was observed during summer in both breeds (Figure 1a,b; Table S1). The Shannon diversity index was higher in spring and summer than in winter, regardless of breed (Figure 1c; Table S1). Out of 3480 observed OTUs, 899 OTUs were shared among all groups (Figure 2). Moreover, 1363 identical OTUs were observed in the steers at different seasons; however, the highest identical OTUs were observed in winter seasons in both breeds compared to other seasons. The PCoA plot showed that overall rumen microbiota was shifted over the seasons in both breeds (Figure 3). In particular, overall rumen microbiota structure in winter was different from those of the other seasons; however, variation between spring and summer was also observed in the PCoA plot regardless of breed.

**Figure 4:**
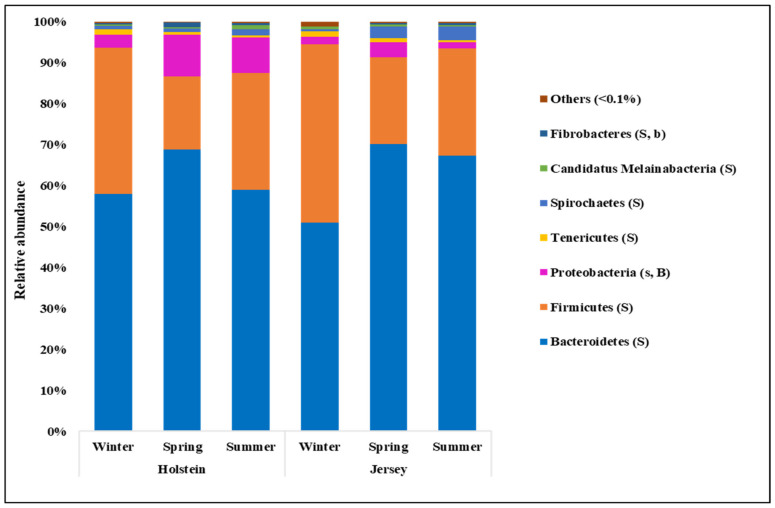
Relative abundance of identified rumen microbial phyla of Holstein and Jersey steers at different seasons. S, and B indicate significant (*p* < 0.05) difference while s, and b indicate tentatively significant (0.05 < *p* < 0.1) difference in relative abundance between seasons, and breeds, respectively.

**Figure 5:**
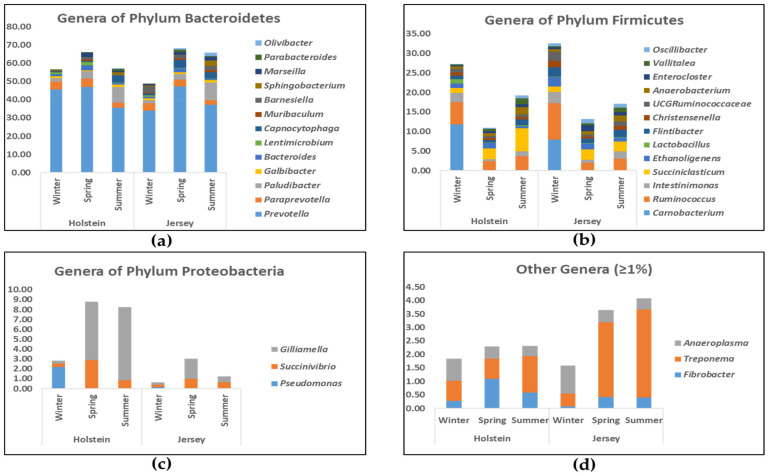
Major genera of bacteria had relative abundance ≥ 1% at least in one breed at one season. (**a**) Genera of phylum Bacteroidetes; (**b**) Genera of phylum Firmicutes; (**c**) Genera of phylum Proteobacteria; (**d**) Other Genera ≥ 1%.

At the phylum level, Bacteroidetes (accounting for 50.96% to 70.08%) and Firmicutes (17.84% to 43.53%) were the two major bacterial taxa across seasons and breeds (Figure 4; Table S2). However, the relative abundance of Bacteroidetes was significantly higher in spring and summer, while that of Firmicutes was significantly greater in winter (*p* < 0.01 for both). Proteobacteria, the third-largest phylum, was more predominant in Holstein steers than Jersey steers regardless of season (*p* < 0.01). The relative abundance of Tenericutes was higher in winter compared to summer regardless of breed; however, the opposite was true for Spirochaetes (*p* = 0.04 and *p* = 0.046 for Tenericutes and Spirochaetes, respectively). *Prevotella*, belonging to the phylum Bacteroidetes, was the most predominant bacterial genus in all seasons, varying from 35.60% to 47.11%; however, the value was significantly higher in spring (*p* = 0.03) compared to other seasons regardless of breed (Figure 5a; Table S3). *Carnobacterium*, the second predominant bacterial genus (*p* < 0.05), was observed only in winter regardless of breed (*p* < 0.01) (Figure 5b; Table S3). *Ruminococcus* and *Intestinimonas* were more abundant bacterial genera in winter than in the other seasons regardless of breed (*p* < 0.01 for both). In contrast, *Paludibacter* was more abundant, while *Paraprevotella* was the less abundant bacterial genera in the summer compared to other seasons irrespective of breed (*p* < 0.01 for *Paludibacter* and *p* = 0.049 for *Paraprevotella*). The relative abundance of the genus *Treponema* was tentatively higher in summer compared to winter regardless of breed (*p* = 0.05) (Figure 5d; Table S3). Furthermore, *Succinivibrio* and *Gilliamella* were more abundant genera in Holstein steers, while *Capnocytophaga*, *Muribaculum*, *Barnesiella*, *Flintibacter*, *UCG*_*Ruminococcaceae*, *Enterocloster*, and *Oscillibacter* were more abundant in Jersey steers regardless of seasons (*p* < 0.05) (Figure 5a–c; Table S3).

At the species level, a total of 19 bacterial species were identified, each with a relative abundance of ≥2% in at least one season in one breed (Table 5). Of these, *P. ruminicola* was the most abundant species but not influenced either by season or breed (*p* > 0.05). However, *P. brevis*, *P. copri*, and *Succinivibrio dextrinosolvens* were more abundant, whereas *Flintibacter Butyricus* was less abundant; *Intestinimonas butyriciproducens* was the least abundant species in the spring compared to the other seasons regardless of breed (*p* ≤ 0.01 for all). The *C. jeotgali*, the second most abundant bacterial species, was observed only in winter regardless of breed (*p* < 0.01). In addition, *R. bromii* was more abundant, while *M. massiliensis* and *Gilliamella bombicola* were less abundant in winter than in the other seasons (*p* < 0.01). *Pal. propionicigenes* was more abundant, while *E. harbinense* was less abundant in summer compared to other seasons regardless of breed (*p* < 0.01 and *p* = 0.03, respectively). Furthermore, *S. dextrinosolvens* and *G. bombicola* were more abundant in Holstein steers, whereas *C. cynodegmi, Barnesiella viscericola*, and *Fl. butyricus* were more abundant in Jersey steers regardless of season (*p* < 0.05). In addition, 21 bacterial species were identified, each with a relative abundance of ≥ 1% (but <2%) in at least one season in one breed (Table 6). Among these, *Anaerobacterium chartisolvens*, *Vallitalea pronyensis*, and *Treponema saccharophilum* were more abundant in the summer, while *P. oris* and *Bacteroides clarus* were more abundant in spring compared to other seasons irrespective of breed (*p* ≤ 0.01 for all). On the other hand, *B. clarus* was more abundant in Holstein steers, while *Enterocloster asparagiformis*, *O. ruminantium*, *Clostridium methylpentosum*, and *T. ruminis* were more abundant in Jersey steers regardless of season (*p* < 0.05).

## 4. Discussion

Both breed and season can affect the growth, rumen fermentation, methane emissions, feed utilization, and other animal productivity traits of ruminants in an age-dependent manner [25,26,30,31,32]. Many studies have compared and evaluated the difference between breeds and seasons [27,36,53]. The present study compared and examined how breed and season might affect the growth performance, rumen fermentation characteristics, methane emissions, and the rumen microbiota using both Holstein and Jersey steers as animal models. To eliminate age and diet as confounding factors, all the animals used in the present study had the same age and consumed the same TMR. The results of this study provided some basic information on the effects of host genetics and physiology on some of the important traits of ruminants.

As expected, Holstein steers had higher total DMI than Jersey steers, which agrees with the results of Flay et al. [54] who reported that the heavier breed of Holstein heifers had higher DMI than their Jersey counterparts. However, Holstein steers had the lowest metabolic DMI in summer, while the lowest total DMI of Jersey steers was observed in winter. This might be due to the higher and lower THI recorded during summer and winter, leading to heat and cold sensitivity of Holstein and Jersey steers, respectively [13]. It was reported earlier that seasonal changes influenced dietary composition and intake of grazing beef steers [55,56]; however, this study offered the same TMR throughout the feeding trial, which indicates the seasonal influence of intake of feedlot steers. Ruminal pH decreases with an increase in VFA production by microbial fermentation or decreases in VFA absorption via the ruminal epithelium or saliva secretion [57]. In the present study, the significantly lower pH observed in summer may be due to decrease in saliva secretion and increase in saliva drooling, which is often observed in animals exposed to high THI [58]. However, the gradual decrease in the concentrations of total VFA and propionate proportion concomitant with the increase in THI may be associated with disturbances in microbial activity in the rumen during summer, which was supported by decrease in OTU abundances in summer, as observed in this study. Earlier studies also reported a significant decrease in VFA production during heat stress conditions [59,60,61]. In contrast, butyrate absorption through the rumen epithelium could greatly decrease under heat stress [62], which corroborates the significantly higher butyrate concentration observed in the present study during high THI in summer. Both seasons and breeds also had a significant influence on the A:P ratio. Holstein steers had a lower A:P ratio, and the lowest value occurred in winter. This might be due to the significantly higher propionate production in winter and by Holstein steers in the current study. Rumen NH_3_-N concentrations can be influenced by dietary protein breakdown, NH_3_ utilization by rumen microbes, absorption by rumen wall, and urea hydrolysis in the rumen [63,64,65]. The highest NH_3_-N concentration observed in summer might be explained by the decrease in its absorption by the rumen wall and utilization by rumen microbes, which may have been greatly affected by high THI during summer. However, the differences in rumen NH_3_-N concentration between the Jersey and Holstein steers could also be attributed to variation in rumen microbiota in the two breeds.

It was hypothesized that Holstein steers would produce more CH_4_ owing to their higher DMI, while Jersey steers should yield less CH_4_ due to their greater feed efficiency [66]. However, we observed numerically higher CH_4_ production and significantly higher CH_4_ yield and intensity in Jersey steers than in Holstein steers. This finding is in agreement with the results of Olijhoek et al. [67], who reported that CH_4_ yield was significantly higher in Jersey cows than in Holstein cows. Propionate-producing rumen microbes compete with methanogens to metabolize H_2_, thereby lowering methane production [68,69]. The lower CH_4_ emissions observed in the Holstein steers could be due to the significantly higher propionate production compared to Jersey steers. In addition, the A:P ratio was higher in high CH_4_ producing Jersey dairy cows [67]. Likewise, our study showed a higher A:P ratio in high CH_4_-producing Jersey steers than in Holstein steers regardless of the season. However, the significantly lower A:P ratio in the winter season might be due to the significantly higher rate of propionate production.

The rumen microbiota responds to variations in host genetics [70], physiological status [25], and diet, among other factors [32]. Its seasonal variation independent of alteration of diet has not been well studied. In the present study, we comparatively examine how season affect the rumen microbiota in both Holstein and Jersey steers fed the identical TMR. Spring witnessed the highest number of observed OTUs and highest Chao 1 richness estimate compared to other seasons; however, summer had highly unstable values irrespective of breed. These results suggest that the rumen microbiota was rich in species when animals are free of stress during spring but that the higher THI in summer could lead to heat stress and significantly affect species richness of the rumen microbiota. The highest Shannon diversity index, which is determined by both species richness and evenness, recorded in the summer season suggests that the rumen environment of heat-stressed steers is more suitable for the proliferation of diverse group of microbes. However, the lowest diversity in the winter season might be attributed to the selective proliferation of microbes mostly associated with high metabolic heat production, which is necessary to maintain homeothermy. The PCoA showed that the composition of the rumen microbiota during winter was different from that of the other seasons. Moreover, seasonal shifting of overall rumen microbiome was observed between spring and summer. The above-mentioned findings confirm seasonal influence on the rumen microbiota in this study. Seasonal shifting of the rumen microbiota has been reported in grazing dairy cows by Noel et al. [36], but the changes in pastures confounded any potential seasonal effect. Martinez-Fernandez et al. [53] also observed that the bacterial community of grazing cattle had changed at mid-dry and wet season with or without a Nitrogen-based supplement. However, the differences in nutrient contents of grazing pastures at different seasons does not represent the seasonal influence of feedlot cattle with same TMR. Usually, cattle alter their energy requirement and body physiology along with the increase and decrease in ambient temperature through a variety of mechanisms. During heat stress, animal core body temperature increases about 1 °C and cattle start thermal homeostasis by increasing sweating, panting, and respiration rate phenotypically. Cattle stimulate the appetite center to reduce feed intake, which leads to reduced rumen motility. Moreover, decreased pH, ruminal absorption of fermented products, and increased rumen temperature change the rumen environment [58,71,72,73]. In contrast, cold stressed-cattle require more energy to maintain homeothermy, which is primarily achieved by increased feed intake and more metabolic heat production [3]. Though the rumen microbiome is the key player in the rumen ecosystem, we hypothesized that the dominancy of rumen microbes might change along with the alteration of rumen environment in different season. Bacteroidetes and Firmicutes are the most abundant group of bacteria in ruminants [74,75,76,77,78,79]. Similarly, in the current study, Bacteroidetes and Firmicutes were the most abundant bacterial phyla in both steers regardless of season; however, the highest relative abundance of Bacteroidetes was recorded in spring and summer, while that of Firmicutes was recorded in winter. Likewise, seasonal variation of some other phyla was seen to exist in this study. These variations in the relative abundance suggest that the rumen ecosystem might be altered according to seasonal changes. Previous studies reported that *Prevotella* was the most abundant bacterial genus in ruminants [77,78,79]. Similarly, in the present study, *Prevotella* was the most abundant bacterial genus in all seasons; however, the relative abundance of this genus was significantly higher than that of *P. brevis* and *P. copri*, which were observed in spring, suggesting their preferential growth in the rumen of steers during spring with normal THI. The *C. jeotgali* can metabolize various carbohydrates as energy sources [80]. In the present study, the genus *Carnobacterium* and the species *C. jeotgali* were only found in winter with a higher percentage of relative abundance, which might be associated with higher VFA production in winter. The species *R. bromii*, belonging to the genus *Ruminococcus*, family *Ruminococcaceae* and phylum Firmicutes, is a starch degrading bacteria present in the rumen [81,82]. The higher abundance of *R. bromii* in winter suggests higher amylolytic activity through their preferential growth in the rumen, which might be attributed to higher VFA production in winter. Baek et al. [83] revealed that heat stress reduced the abundance of fibrolytic Ruminococcaceae while increasing the lactate-producing Lactobacillaceae and amylolytic *Prevotella* and *Ruminobacter* in Hanwoo steers. Likewise, Zhao et al. [84] reported that heat-stressed dairy cows had a significantly higher relative abundance of *Streptococcus*, unclassified *Enterobacteriaceae*, *Ruminobacter*, *Treponema*, and unclassified Bacteroidaceae. Similarly, in the present study, both steers had higher relative abundance of *Treponema, Paludibacter*, *Pal. propionicigenes* during summer, suggesting their suitable growth environment in the rumen of steers during high THI. Moreover, both steers had some other distinct bacterial genera and species with higher relative abundance in different seasons, further confirming the seasonal influence. Therefore, rumen microbial richness, diversity, and community composition were greatly altered according to seasonal stress, either cold or heat, even when cattle were fed the same TMR.

Holstein and Jersey steers did not differ in numbers of observed OTUs, Chao 1 richness estimate, Shannon, and inverse Simpson diversity indexes, which is in contrast to the report of Paz et al. [27], which reported significantly higher alpha diversity metrics, including Chao1 richness estimates and the number of observed OTUs, in Holstein cows than in lactating Jersey cows. This discrepancy may be due to the variation in the microbiota affecting the host genetics and other factors, especially sex [70,85] and physiological state [86]. Similar to seasons, Bacteroidetes and Firmicutes were the most predominant bacterial phyla in the rumen of both the Holstein and Jersey steers but did not differ significantly between breeds. The relative abundances of the phyla Proteobacteria, the genera *Succinivibrio* and *Gilliamella*, and the species *S. dextrinosolvens*, *G. bombicola*, *B. clarus*, and *P. enoeca* were higher in the Holstein steers, while the genera *Flintibacter*, *Barnesiella*, *Capnocytophaga*, UCG_*Ruminococcaceae*, *Enterocloster*, and *Oscillibacter*, and the species *Cap. cynodegmi*, *Fl. Butyricus*, *O. ruminantium*, and *Clostridium methylpentosum* were more abundant in Jersey steers, suggesting their preferential growth in the rumen of particular breeds. *Fl. Butyricus* and *O. ruminantium* can produce butyrate from carbohydrates [87,88]. In our study, a significantly higher butyrate concentration was observed in Jersey steers, which may be associated with the higher relative abundance of these bacteria in this breed. Several species of the genus *Succinivibrio*, *S. dextrinosolvens* in particular, produce succinate, which can subsequently be converted to propionate by propionate-producing bacteria [89,90]. In the present study, Holstein steers had a significantly higher relative abundance of *S. dextrinosolvens* than Jersey steers, which was consistent with the relative propionate concentrations and CH_4_ emissions in the two breeds. The differences in the above-mentioned microbial abundances suggest that breed has a significant influence on rumen microbial community composition even when the same TMR diet is given.

## 5. Conclusions

Summer reduced metabolic DMI of Holstein, while winter reduced total DMI of Jersey steers regardless of season. While season had no influence on enteric CH_4_ emissions, the breed of steers had significant influence on it. Summer lowered ruminal pH; however, summer increased NH_3_-N concentration irrespective of breed. Winter increased total VFA concentration and propionate proportion, while spring increased acetate and summer increased butyrate proportion regardless of breed. Holstein steers produced more propionate, while Jersey steers produced more butyrate regardless of season. Richness and diversity of the rumen microbiota were shifted according to seasonal changes, even when the same TMR was given. In addition, distinct rumen microbial communities were observed in all seasons and both breeds with high relative abundance, which might influence rumen fermentation. Overall, this study suggests that both seasons and breeds should be taken into consideration during manipulating rumen microbiome to improve rumen fermentations. Moreover, breed specific mitigation approaches are needed to mitigate CH_4_ emissions from the Holstein and Jersey steers. Further analysis of the functional genes of rumen microbiome, as well as metatranscriptomics, is required to reveal the intense relationship among rumen microbes, metabolites, and host responses; this relationship will be considered in the subsequent research.

## Figures and Tables

**Table 1:** Ingredients and chemical composition of the total mixed ration (TMR) fed to the steers.

Ingredients	Compositions (% of DM)
Corn grain	36.80
Corn gluten feed	17.89
Lupin	12.49
Wheat bran	11.61
Oat hay	20.26
Limestone (1 mm size)	0.68
Vitamin premix ^1^	0.07
Mineral premix ^2^	0.07
Salt	0.14
Total	100.00
Chemical composition (% as DM basis)	
DM (% as fed basis)	66.30
Crude protein	17.99
Crude Fiber	12.55
Crude fat	4.44
Ash	7.42
Calcium	0.83
Phosphorous	0.55
NDF	36.18
ADF	16.91
TDN	80.47

^1^ The vitamin premix contained (g/kg) L-ascorbic acid, 121.2; DL-α-tocopherol acetate, 18.8; thiamin hydrochloride, 2.7; riboflavin, 9.1; pyridoxine hydrochloride, 1.8; niacin, 36.4; Ca-D-pantothenate, 12.7; myo-inositol, 181.8; D-biotin, 0.27; folic acid, 0.68; p-aminobenzoic acid, 18.2; menadione, 1.8; retinal acetate, 0.73; cholecalciferol, 0.003; and cyanocobalamin, 0.003; and the remaining was cellulose. ^2^ The mineral premix contained (g/kg) MgSO_4_ · 7H2O, 80.0; NaH_2_PO_4_ · 2H_2_O, 370.0; KCl, 130.0; ferric citrate, 40.0; ZnSO_4_ · 7H_2_O, 20.0; Ca-lactate, 356.5; CuCl_2_, 0.2; AlCl_3_ · 6H_2_O, 0.15; KI, 0.15; Na_2_Se_2_O_3_, 0.01; MnSO_4_ · H2O, 2.0; and CoCl_2_ · 6H_2_O, 1.0. DM, dry matter; NDF, neutral detergent fiber; ADF, acid detergent fiber; TDN, total digestible nutrient.

**Table 2:** The recorded ambient temperature, relative humidity, and Temperature Humidity Index during the study periods.

Seasons	Minimum Ambient Temp. (°C)	Maximum Ambient Temp. (°C)	Mean Ambient Temp. (°C)	rH (%)	THI	Stress Categories
Winter	−0.18 ± 2.58	6.03 ± 2.97	3.19 ± 3.09	53.26 ± 8.14	45.37 ± 3.63	Cold Stress
Spring	5.59 ± 2.27	15.92 ± 2.62	10.30 ± 1.96	53.55 ± 15.12	57.07 ± 2.89	No Stress
Summer	27.02 ± 0.30	31.58 ± 2.31	29.16 ± 1.06	85.15 ± 8.00	84.16 ± 1.64	Heat Stress

rH, relative humidity; THI, temperature humidity index.

**Table 3:** Dry matter intake, growth performance, and methane emissions of Holstein and Jersey steers at different seasons.

Parameters	Breed	Season				SEM	Mixed *p*-Value		
		Winter	Spring	Summer	Overall		Season	Breed	S × B
DMI (kg/d)	Hol	13.42 ^bx^	14.84 ^ax^	12.58 ^bx^	13.61	0.419	<0.01	<0.01	<0.01
	Jer	9.66 ^by^	11.46 ^ay^	12.04 ^ay^	11.05	0.395			
	Total	11.54 ^b^	13.15 ^a^	12.31 ^ab^	-	0.407			
DMI (g/d/Kg BW^0.75^)	Hol	120.33 ^a^	123.45 ^ax^	94.68 ^by^	112.82	2.971	<0.01	0.56	<0.01
	Jer	106.35	116.50 ^y^	110.27 ^x^	111.04	3.417			
	Total	113.34 ^b^	119.98 ^a^	102.47 ^c^	-	3.194			
CH_4_ production (g/d)	Hol	162.42	165.74	129.55	152.57	13.381	0.57	0.11	0.14
	Jer	154.92	180.56	187.30	174.26	15.303			
	Total	158.67	173.15	158.43	-	14.342			
CH_4_ yield (g/d/kg DMI)	Hol	12.93	10.95	10.49	11.46	1.099	0.26	<0.01	0.99
	Jer	18.33	16.40	15.60	16.78	1.696			
	Total	15.63	13.68	13.05	-	1.398			
CH_4_ intensity (g/d/kg BW^0.75^)	Hol	1.47	1.37	0.98	1.28	0.101	0.22	<0.01	0.44
	Jer	1.78	1.90	1.75	1.81	0.203			
	Total	1.63	1.64	1.36	-	0.152			

DMI, dry matter intake; BW^0.75^, metabolic body weight; CH_4_, methane; SEM, standard error of the mean; Hol, Holstein steer; Jer, Jersey steer. ^a,b,c^ in the same row indicate the significant differences (*p* < 0.05) of data among three different seasons of each breed as well as regardless of breed. ^x,y^ in the same column indicate the significant differences (*p* < 0.05) of data between two breeds in each season.

**Table 4:** Rumen fermentation characteristics of Holstein and Jersey steers at different seasons.

Parameters	Breed	Season				SEM	Mixed *p*-Value		
		Winter	Spring	Summer	Overall		Season	Breed	S × B
pH	Hol	6.62	6.64	6.50	6.59	0.055	0.02	0.03	0.49
	Jer	6.59	6.47	6.37	6.48	0.061			
	Total	6.60 ^a^	6.56 ^a^	6.44 ^b^	-	0.058			
NH_3_-N (mg/dL)	Hol	3.19	3.07	5.06	3.77	0.322	<0.01	0.03	0.22
	Jer	3.96	4.38	5.06	4.77	0.453			
	Total	3.57 ^b^	3.73 ^b^	5.06 ^a^	-	0.387			
Total VFA (mmol/L)	Hol	103.34	92.93	91.01	95.76	1.574	<0.01	0.64	0.80
	Jer	102.83	94.65	91.73	96.40	1.514			
	Total	103.08 ^a^	93.79 ^b^	91.37 ^c^	-	1.544			
Acetate (mol/100 mol)	Hol	62.18	64.99	63.54	63.57	0.617	<0.01	0.56	0.91
	Jer	62.73	65.13	63.66	63.84	0.441			
	Total	62.46 ^b^	65.06 ^a^	63.60 ^b^	-	0.529			
Propionate (mol/100 mol)	Hol	25.46	20.67	20.16	22.10	0.470	<0.01	<0.01	0.58
	Jer	23.15	18.96	18.80	20.31	0.415			
	Total	24.31 ^a^	19.82 ^b^	19.48 ^b^	-	0.442			
Butyrate (mol/100 mol)	Hol	12.35	14.34	16.30	14.33	0.324	<0.01	<0.01	0.68
	Jer	14.12	15.91	17.54	15.86	0.275			
	Total	13.24 ^c^	15.13 ^b^	16.92 ^a^	-	0.300			
A:P	Hol	2.47	3.15	3.17	2.93	0.079	<0.01	<0.01	0.89
	Jer	2.72	3.46	3.40	3.19	0.086			
	Total	2.59 ^b^	3.30 ^a^	3.28 ^a^	-	0.082			

NH_3_-N, ammonia-nitrogen; VFA, volatile fatty acids; A:P, acetate: propionate ratio; SEM, standard error of the mean; Hol, Holstein steer; Jer, Jersey steer. ^a,b,c^ in the same row indicate the significant differences (*p* < 0.05) of data among three different seasons regardless of breed.

**Table 5:** Major species of bacteria had relative abundance ≥2% at least in one breed at one season.

Phylum	Species	Breed	Season				SEM	Mixed *p*-Value	
			Winter	Spring	Summer	Overall		Season	Breed
Bacteroidetes	*Prevotella ruminicola*	Hol	35.83	27.90	26.24	29.99	3.041	0.35	0.36
		Jer	27.99	26.85	27.74	27.53	4.178		
		Total	31.91	27.37	26.99	-	3.609		
	*Paraprevotella clara*	Hol	4.48	4.59	2.72	3.93	0.723	0.07	0.18
		Jer	3.94	3.89	2.24	3.36	0.921		
		Total	4.21	4.24	2.48	-	0.822		
	*Prevotella brevis*	Hol	3.56	7.01	3.20	4.59	0.998	<0.01	0.92
		Jer	4.33	9.75	2.21	5.43	1.423		
		Total	3.95 ^b^	8.38 ^a^	2.70 ^b^	-	1.210		
	*Paludibacter propionicigenes*	Hol	1.81	4.06	8.63	4.83	1.408	<0.01	0.41
		Jer	1.04	2.93	9.50	4.49	1.182		
		Total	1.42 ^b^	3.49 ^b^	9.06 ^a^	-	1.295		
	*Prevotella oralis*	Hol	0.78	1.42	0.56	0.92	0.386	0.58	0.95
		Jer	1.41	0.72	2.68	1.60	0.922		
		Total	1.10	1.07	1.62	-	0.654		
	*Capnocytophaga cynodegmi*	Hol	0.60	0.41	3.25	1.42	0.437	0.02	0.03
		Jer	0.94	4.27	3.23	2.81	0.918		
		Total	0.77 ^b^	2.3^ab^	3.24 ^a^	-	0.677		
	*Prevotella copri*	Hol	0.37	4.18	1.49	2.01	0.323	<0.01	0.26
		Jer	0.34	2.85	0.89	1.36	0.433		
		Total	0.35 ^b^	3.51 ^a^	1.19 ^b^	-	0.378		
	*Marseilla massiliensis*	Hol	0.14	2.20	1.03	1.12	0.499	<0.01	0.30
		Jer	0.33	1.30	1.85	1.16	0.298		
		Total	0.23 ^b^	1.75 ^a^	1.44 ^a^	-	0.399		
	*Barnesiella viscericola*	Hol	0.05	0.21	0.21	0.16	0.042	0.94	0.01
		Jer	3.08	1.08	0.47	1.54	0.578		
		Total	1.56	0.65	0.34	-	0.310		
	*Sphingobacterium daejeonense*	Hol	0.02	0.01	0.99	0.34	0.312	0.14	0.34
		Jer	0.21	0.05	2.96	1.07	0.832		
		Total	0.11	0.03	1.98	-	0.572		
Firmicutes	*Carnobacterium jeotgali*	Hol	11.83	0.00	0.00	3.94	0.554	<0.01	0.47
		Jer	8.72	0.00	0.00	2.91	1.456		
		Total	10.27 ^a^	0.00 ^b^	0.00 ^b^	-	1.005		
	*Ruminococcus bromii*	Hol	4.10	0.86	1.51	2.16	0.581	<0.01	0.66
		Jer	5.26	0.91	1.41	2.53	0.635		
		Total	4.68 ^a^	0.89 ^b^	1.46 ^b^	-	0.608		
	*Intestinimonas butyriciproducens*	Hol	2.43	0.55	1.27	1.42	0.250	<0.01	0.28
		Jer	2.98	0.69	1.89	1.85	0.310		
		Total	2.71 ^a^	0.62 ^c^	1.58 ^b^	-	0.280		
	*Succiniclasticum ruminis*	Hol	1.30	2.80	5.83	3.31	1.036	<0.01	0.28
		Jer	0.87	2.70	2.50	2.02	0.613		
		Total	1.08 ^b^	2.75^ab^	4.17 ^a^	-	0.824		
	*Ethanoligenens harbinense*	Hol	1.12	1.31	0.61	1.01	0.193	0.03	0.57
		Jer	2.28	1.43	1.01	1.57	0.678		
		Total	1.70 ^a^	1.37 ^a^	0.81 ^b^	-	0.435		
	*Flintibacter butyricus*	Hol	0.95	0.61	1.46	1.01	0.179	0.01	0.01
		Jer	2.37	1.10	1.98	1.82	0.333		
		Total	1.66 ^a^	0.86 ^b^	1.72 ^a^	-	0.256		
	*Ruminococcus albus*	Hol	0.70	0.64	1.15	0.83	0.185	0.10	0.83
		Jer	2.10	0.58	0.86	1.18	0.323		
		Total	1.40	0.61	1.00	-	0.254		
Proteobacteria	*Succinivibrio dextrinosolvens*	Hol	0.36	2.87	0.86	1.36	0.257	0.01	0.01
		Jer	0.29	0.98	0.67	0.65	0.411		
		Total	0.32 ^b^	1.92 ^a^	0.77 ^b^	-	0.334		
	*Gilliamella bombicola*	Hol	0.29	5.93	7.41	4.54	1.772	<0.01	0.02
		Jer	0.22	2.07	0.57	0.95	0.451		
		Total	0.26 ^b^	4.00 ^a^	3.99 ^a^	-	1.112		

SEM, standard error of the mean; Hol, Holstein steer; Jer, Jersey steer. ^a,b,c^ in the same row indicate the significant differences (*p* < 0.05) of data among three different seasons regardless of breed.

**Table 6:** Species of bacteria had relative abundance ≥ 1% but <2% at least in one breed at one season.

Phylum	Species	Breed	Season				SEM	Mixed *p*-Value	
			Winter	Spring	Summer	Overall		Season	Breed
Bacteroidetes	*Barnesiella intestinihominis*	Hol	0.22	1.20	0.29	0.57	0.271	<0.01	0.31
		Jer	0.12	1.07	1.92	1.03	0.531		
		Total	0.17 ^b^	1.13 ^a^	1.10 ^ab^	-	0.401		
	*Lentimicrobium saccharophilum*	Hol	0.85	1.87	0.24	0.98	0.483	0.01	0.78
		Jer	0.75	0.40	0.33	0.50	0.091		
		Total	0.80 ^ab^	1.14 ^a^	0.28 ^b^	-	0.287		
	*Galbibacter mesophilus*	Hol	1.21	0.65	1.38	1.08	0.204	0.09	0.98
		Jer	1.09	0.96	1.30	1.12	0.260		
		Total	1.15	0.81	1.34	-	0.232		
	*Muribaculum intestinale*	Hol	0.31	0.80	0.18	0.43	0.189	0.11	0.07
		Jer	0.42	0.70	1.04	0.72	0.319		
		Total	0.36	0.75	0.61	-	0.254		
	*Prevotella micans*	Hol	0.74	0.54	1.00	0.76	0.314	0.01	0.33
		Jer	0.32	1.14	1.48	0.98	0.270		
		Total	0.53 ^b^	0.84 ^ab^	1.24 ^a^	-	0.292		
	*Prevotella oris*	Hol	0.47	1.96	0.36	0.93	0.207	<0.01	0.09
		Jer	0.29	0.99	0.22	0.50	0.146		
		Total	0.38 ^b^	1.47 ^a^	0.29 ^b^	-	0.177		
	*Bacteroides clarus*	Hol	0.75	1.75	0.40	0.96	0.264	0.01	0.04
		Jer	0.11	1.28	0.37	0.58	0.258		
		Total	0.43 ^b^	1.51 ^a^	0.38 ^b^	-	0.261		
	*Prevotella enoeca*	Hol	1.36	0.00	0.02	0.46	0.457	0.02	0.03
		Jer	0.00	0.00	0.00	0.00	0.001		
		Total	0.68 ^a^	0.00 ^b^	0.01 ^b^	-	0.229		
	*Olivibacter sitiensis*	Hol	0.00	0.00	0.00	0.00	0.001	0.02	0.13
		Jer	0.00	0.73	1.83	0.85	0.693		
		Total	0.00 ^b^	0.36 ^ab^	0.92 ^a^	-	0.347		
Candidatus Melainabacteria	*Vampirovibrio chlorellavorus*	Hol	0.39	0.37	1.07	0.61	0.149	0.04	0.95
		Jer	0.62	0.37	0.48	0.49	0.096		
		Total	0.50 ^ab^	0.37 ^b^	0.77 ^a^	-	0.122		
Fibrobacteres	*Fibrobacter succinogenes*	Hol	0.28	1.09	0.58	0.65	0.232	<0.01	0.07
		Jer	0.07	0.43	0.40	0.30	0.081		
		Total	0.17 ^b^	0.76 ^a^	0.49 ^ab^	-	0.157		
Firmicutes	*Christensenella massiliensis*	Hol	0.61	0.42	0.41	0.48	0.108	0.01	0.43
		Jer	1.04	0.38	0.47	0.63	0.121		
		Total	0.82 ^a^	0.40 ^b^	0.44 ^b^	-	0.114		
	*Anaerobacterium chartisolvens*	Hol	0.63	0.67	1.87	1.06	0.240	0.01	0.73
		Jer	0.61	0.78	1.45	0.95	0.258		
		Total	0.62 ^b^	0.73 ^b^	1.66 ^a^	-	0.249		
	*Vallitalea pronyensis*	Hol	0.20	0.62	1.47	0.76	0.142	<0.01	0.66
		Jer	0.31	0.53	1.04	0.62	0.146		
		Total	0.26 ^b^	0.57 ^b^	1.26 ^a^	-	0.144		
	*Enterocloster asparagiformis*	Hol	0.29	0.56	0.77	0.54	0.095	<0.01	0.04
		Jer	0.36	1.53	0.91	0.93	0.135		
		Total	0.32 ^b^	1.04 ^a^	0.84 ^a^	-	0.115		
	*Oscillibacter ruminantium*	Hol	0.15	0.18	0.74	0.36	0.090	0.01	0.01
		Jer	0.71	1.10	0.98	0.93	0.307		
		Total	0.43 ^b^	0.64 ^ab^	0.86 ^a^	-	0.199		
	*Clostridium methylpentosum*	Hol	0.29	0.27	0.22	0.26	0.082	0.13	0.02
		Jer	1.44	0.31	0.49	0.74	0.285		
		Total	0.86	0.29	0.35	-	0.183		
	*Lactobacillus sakei*	Hol	1.01	0.00	0.00	0.34	0.181	<0.01	0.94
		Jer	0.14	0.00	0.00	0.05	0.026		
		Total	0.58 ^a^	0.00 ^b^	0.00 ^b^	-	0.103		
Spirochaetes	*Treponema porcinum*	Hol	0.22	0.20	0.08	0.17	0.077	0.90	0.21
		Jer	0.12	0.18	1.02	0.44	0.214		
		Total	0.17	0.19	0.55	-	0.146		
	*Treponema ruminis*	Hol	0.09	0.08	0.16	0.11	0.044	0.21	0.04
		Jer	0.10	1.20	0.37	0.55	0.214		
		Total	0.10	0.64	0.26	-	0.129		
	*Treponema saccharophilum*	Hol	0.04	0.07	0.41	0.17	0.064	<0.01	0.98
		Jer	0.01	0.19	1.21	0.47	0.233		
		Total	0.03 ^b^	0.13 ^b^	0.81 ^a^	-	0.149		

SEM, standard error of the mean; Hol, Holstein steer; Jer, Jersey steer. ^a,b^ in the same row indicate the significant differences (*p* < 0.05) of data among three different seasons regardless of breed.

## Data Availability

The datasets presented in this study can be found in online repositories. The name of the repository (NCBI) and accession number (PRJNA667610) can be found in the following link: https://www.ncbi.nlm.nih.gov/sra/PRJNA667610.

## Supplementary Materials

The following are available online at https://www.mdpi.com/article/10.3390/ani11041184/s1, Table S1: Richness and diversity of rumen microbiome of Holstein and Jersey steers at different seasons, Table S2: Major bacterial phyla identified in the rumen of the Holstein and the Jersey steers at different seasons, Table S3: Major genera of bacteria had relative abundance ≥ 1% at least in one breed at one season.

## References

[1] Intergovernmental Panel on Climate Change (2014). Climate Change 2014 Synthesis Report—IPCC.

[2] Rojas-Downing M.M., Nejadhashemi A.P., Harrigan T., Woznicki S.A. (2017). Climate change and livestock: Impacts, adaptation, and mitigation. Clim. Risk Manag..

[3] Ames D.R., Brink D.R., Willms C.L. (1980). Adjusting Protein in Feedlot Diets during Thermal Stress. J. Anim. Sci..

[4] Birkelo C.P., Johnson D.E., Phetteplace H.P. (1991). Maintenance requirements of beef cattle as affected by season on different planes of nutrition. J. Anim. Sci..

[5] Hahn G.L. (1995). Environmental influences on feed intake and performance of feedlot cattle. Res. Rep. P.

[6] Mader T.L., Fell L.R., McPhee M.J. (1997). Behavior response of non-Brahman cattle to shade in commercial feedlots. Livest. Environ..

[7] Kang H.J., Lee I.K., Piao M.Y., Gu M.J., Yun C.H., Kim H.J., Kim K.H., Baik M. (2016). Effects of ambient temperature on growth performance, blood metabolites, and immune cell populations in Korean cattle steers. Asian Australas. J. Anim. Sci..

[8] Kang H.J., Piao M.Y., Park S.J., Na S.W., Kim H.J., Baik M. (2019). Effects of ambient temperature and rumen—Protected fat supplementation on growth performance, rumen fermentation and blood parameters during cold season in Korean cattle steers. Asian Australas. J. Anim. Sci..

[9] Yadav B., Pandey V., Yadav S., Singh Y., Kumar V., Sirohi R. (2016). Effect of misting and wallowing cooling systems on milk yield, blood and physiological variables during heat stress in lactating Murrah buffalo. J. Anim. Sci. Technol..

[10] Brown-Brandl T.M., Nienaber J.A., Eigenberg R.A., Hahn G.L., Freetly H. (2003). Thermoregulatory responses of feeder cattle. J. Therm. Biol..

[11] Smith D.L., Smith T., Rude B.J., Ward S.H. (2013). Short communication: Comparison of the effects of heat stress on milk and component yields and somatic cell score in Holstein and Jersey cows. J. Dairy Sci..

[12] Kim D.H., Kim M.H., Kim S.B., Son J.K., Lee J.H., Joo S.S., Gu B.H., Park T., Park B.Y., Kim E.T. (2020). Differential dynamics of the ruminal microbiome of jersey cows in a heat stress environment. Animals.

[13] Aggarwal A., Upadhyay R. (2013). Heat stress and animal productivity. Heat Stress and Animal Productivity.

[14] Barton R.A., Donaldson J.L., Barnes F.R., Jones C.F., Clifford H.J. (1994). Comparison of friesian, friesian-jersey-cross, and jersey steers in beef production. N. Z. J. Agric. Res..

[15] Schaefer D.M. (2005). Yield and quality of Holstein beef. Manag. Mark. Qual. Holst. Steers Proc..

[16] Mamuad L.L., Lee S.S., Lee S.S. (2019). Recent insight and future techniques to enhance rumen fermentation in dairy goats. Asian Australas. J. Anim. Sci..

[17] Hook S.E., Wright A.D.G., McBride B.W. (2010). Methanogens: Methane producers of the rumen and mitigation strategies. Archaea.

[18] Deng W., Xi D., Mao H., Wanapat M. (2008). The use of molecular techniques based on ribosomal RNA and DNA for rumen microbial ecosystem studies: A review. Mol. Biol. Rep..

[19] Islam M., Lee S. (2018). Recent Application Technologies of Rumen Microbiome Is the Key to Enhance Feed Fermentation. J. Life Sci..

[20] Moss A.R., Jouany J.P., Newbold J. (2000). Methane production by ruminants: Its contribution to global warming. Anim. Res..

[21] Ellis J.L., Dijkstra J., Kebreab E., Bannink A., Odongo N.E., McBride B.W., France J. (2008). Aspects of rumen microbiology central to mechanistic modelling of methane production in cattle. J. Agric. Sci..

[22] Islam M., Lee S.-S. (2019). Advanced estimation and mitigation strategies: A cumulative approach to enteric methane abatement from ruminants. J. Anim. Sci. Technol..

[23] Johnson K.A., Johnson D.E. (1995). Methane emissions from cattle. J. Anim. Sci..

[24] Appuhamy J.A.D.R.N., France J., Kebreab E. (2016). Models for predicting enteric methane emissions from dairy cows in North America, Europe, and Australia and New Zealand. Glob. Chang. Biol..

[25] Zhu Z., Noel S.J., Difford G.F., Al-Soud W.A., Brejnrod A., Sørensen S.J., Lassen J., Løvendahl P., Højberg O. (2017). Community structure of the metabolically active rumen bacterial and archaeal communities of dairy cows over the transition period. PLoS ONE.

[26] Uyeno Y., Shigemori S., Shimosato T. (2015). Effect of probiotics/prebiotics on cattle health and productivity. Microbes Environ..

[27] Paz H.A., Anderson C.L., Muller M.J., Kononoff P.J., Fernando S.C. (2016). Rumen bacterial community composition in holstein and jersey cows is different under same dietary condition and is not affected by sampling method. Front. Microbiol..

[28] O’Hara E., Neves A.L.A., Song Y., Guan L.L. (2020). The Role of the Gut Microbiome in Cattle Production and Health: Driver or Passenger?. Annu. Rev. Anim. Biosci..

[29] Miguel M., Mamuad L., Ramos S., Ku M.J., Jeong C.D., Kim S.H., Cho Y.I., Lee S.S. (2020). Effects of using different roughages in the TMR inoculated with or without coculture of *Lactobacillus acidophilus* and *Bacillus subtilis* on in vitro rumen fermentation and microbial population. Asian Australas. J. Anim. Sci..

[30] Malmuthuge N., Guan L.L. (2017). Understanding host-microbial interactions in rumen: Searching the best opportunity for microbiota manipulation. J. Anim. Sci. Biotechnol..

[31] Jami E., Israel A., Kotser A., Mizrahi I. (2013). Exploring the bovine rumen bacterial community from birth to adulthood. ISME J..

[32] Hua C., Tian J., Tian P., Cong R., Luo Y., Geng Y., Tao S., Ni Y., Zhao R. (2017). Feeding a high concentration diet induces unhealthy alterations in the composition and metabolism of ruminal microbiota and host response in a goat model. Front. Microbiol..

[33] Biswas A.A., Lee S.S., Mamuad L.L., Kim S.H., Choi Y.J., Bae G.S., Lee K., Sung H.G., Lee S.S. (2016). Use of lysozyme as a feed additive on in vitro rumen fermentation and methane emission. Asian Australas. J. Anim. Sci..

[34] Biswas A.A., Lee S.S., Mamuad L.L., Kim S.H., Choi Y.J., Lee C., Lee K., Bae G.S., Lee S.S. (2018). Effects of illite supplementation on in vitro and in vivo rumen fermentation, microbial population and methane emission of Hanwoo steers fed high concentrate diets. Anim. Sci. J..

[35] Li H., Li R., Chen H., Gao J., Wang Y., Zhang Y., Qi Z. (2020). Effect of different seasons (spring vs. summer) on the microbiota diversity in the feces of dairy cows. Int. J. Biometeorol..

[36] Noel S.J., Attwood G.T., Rakonjac J., Moon C.D., Waghorn G.C., Janssen P.H. (2017). Seasonal changes in the digesta-adherent rumen bacterial communities of dairy cattle grazing pasture. PLoS ONE.

[37] AOAC (2005). Official Methods of Analysis of the Association of Official Analytical Chemists.

[38] Van Soest P.J., Robertson J.B., Lewis B.A. (1991). Methods for Dietary Fiber, Neutral Detergent Fiber, and Nonstarch Polysaccharides in Relation to Animal Nutrition. J. Dairy Sci..

[39] Van Soest P. (1973). Collaborative study of acid-detergent fiber and lignin. J. Assoc. Off. Anal. Chem..

[40] Davis M.S., Mader T.L., Holt S.M., Parkhurst A.M. (2003). Strategies to reduce feedlot cattle heat stress: Effects on tympanic temperature. J. Anim. Sci..

[41] Hammond K.J., Humphries D.J., Crompton L.A., Green C., Reynolds C.K. (2015). Methane emissions from cattle: Estimates from short-term measurements using a GreenFeed system compared with measurements obtained using respiration chambers or sulphur hexafluoride tracer. Anim. Feed Sci. Technol..

[42] Hristov A.N., Oh J., Giallongo F., Frederick T., Weeks H., Zimmerman P.R., Harper M.T., Hristova R.A., Zimmerman R.S., Branco A.F. (2015). The use of an automated system (GreenFeed) to monitor enteric methane and carbon dioxide emissions from ruminant animals. J. Vis. Exp..

[43] Chaney A.L., Marbach E.P. (1962). Modified reagents for determination of urea and ammonia. Clin. Chem..

[44] Han S.K., Kim S.H., Shin H.S. (2005). UASB treatment of wastewater with VFA and alcohol generated during hydrogen fermentation of food waste. Process Biochem..

[45] Tabaru H., Kadota E., Yamada H., Sasaki N., Takeuchi A. (1988). Determination of volatile fatty acids and lactic acid in bovine plasma and ruminal fluid by high performance liquid chromatography. Jpn. J. Vet. Sci..

[46] Claassen S., du Toit E., Kaba M., Moodley C., Zar H.J., Nicol M.P. (2013). A comparison of the efficiency of five different commercial DNA extraction kits for extraction of DNA from faecal samples. J. Microbiol. Methods.

[47] Klindworth A., Pruesse E., Schweer T., Peplies J., Quast C., Horn M., Glöckner F.O. (2013). Evaluation of general 16S ribosomal RNA gene PCR primers for classical and next-generation sequencing-based diversity studies. Nucleic Acids Res..

[48] Bolger A.M., Lohse M., Usadel B. (2014). Trimmomatic: A flexible trimmer for Illumina sequence data. Bioinformatics.

[49] Magoč T., Salzberg S.L. (2011). FLASH: Fast length adjustment of short reads to improve genome assemblies. Bioinformatics.

[50] Li W., Fu L., Niu B., Wu S., Wooley J. (2012). Ultrafast clustering algorithms for metagenomic sequence analysis. Brief. Bioinform..

[51] Zhang Z., Schwartz S., Wagner L., Miller W. (2000). A greedy algorithm for aligning DNA sequences. J. Comput. Biol..

[52] SAS Institute (2013). SAS Statistical Analysis Systems for Windows.

[53] Martinez-Fernandez G., Jiao J., Padmanabha J., Denman S.E., McSweeney C.S. (2020). Seasonal and Nutrient Supplement Responses in Rumen Microbiota Structure and Metabolites of Tropical Rangeland Cattle. Microorganisms.

[54] Flay H.E., Kuhn-Sherlock B., Macdonald K.A., Camara M., Lopez-Villalobos N., Donaghy D.J., Roche J.R. (2019). Hot topic: Selecting cattle for low residual feed intake did not affect daily methane production but increased methane yield. J. Dairy Sci..

[55] Cline H.J., Neville B.W., Lardy G.P., Caton J.S. (2009). Influence of advancing season on dietary composition, intake, site of digestion, and microbial efficiency in beef steers grazing a native range in western North Dakota. J. Anim. Sci..

[56] Cline H.J., Neville B.W., Lardy G.P., Caton J.S. (2010). Influence of advancing season on dietary composition, intake, site of digestion, and microbial efficiency in beef steers grazing season-long or twice-over rotation native range pastures in western North Dakota. J. Anim. Sci..

[57] Aikman P.C., Henning P.H., Humphries D.J., Horn C.H. (2011). Rumen pH and fermentation characteristics in dairy cows supplemented with *Megasphaera elsdenii* NCIMB 41125 in early lactation. J. Dairy Sci..

[58] Mader T.L., Davis M.S., Brown-Brandl T. (2006). Environmental factors influencing heat stress in feedlot cattle. J. Anim. Sci..

[59] Moody E.G., van Soest P.J., McDowell R.E., Ford G.L. (1967). Effect of High Temperature and Dietary Fat on Performance of Lactating Cows. J. Dairy Sci..

[60] Tajima K., Nonaka I., Higuchi K., Takusari N., Kurihara M., Takenaka A., Mitsumori M., Kajikawa H., Aminov R.I. (2007). Influence of high temperature and humidity on rumen bacterial diversity in Holstein heifers. Anaerobe.

[61] Yadav B., Singh G., Verma A.K., Dutta N., Sejian V. (2013). Impact of heat stress on rumen functions. Vet. World.

[62] Bedford A., Beckett L., Harthan L., Wang C., Jiang N., Schramm H., Guan L.L., Daniels K.M., Hanigan M.D., White R.R. (2020). Ruminal volatile fatty acid absorption is affected by elevated ambient temperature. Sci. Rep..

[63] Tamminga S. (1979). Protein Degradation in the Forestomachs of Ruminants. J. Anim. Sci..

[64] Wallace R.J. (1996). Conference: Altering Ruminai Nitrogen Metabolism to Improve Protein utilization Ruminai Microbial Metabolism of Peptides and Amino Acids. J. Nutr..

[65] Firkins J.L., Yu Z., Morrison M. (2007). Ruminal nitrogen metabolism: Perspectives for integration of microbiology and nutrition for dairy. J. Dairy Sci..

[66] Spaans O.K., Macdonald K.A., Lancaster J.A.S., Bryant A.M., Roche J.R. (2018). Dairy cow breed interacts with stocking rate in temperate pasture-based dairy production systems. J. Dairy Sci..

[67] Olijhoek D.W., Løvendahl P., Lassen J., Hellwing A.L.F., Höglund J.K., Weisbjerg M.R., Noel S.J., McLean F., Højberg O., Lund P. (2018). Methane production, rumen fermentation, and diet digestibility of Holstein and Jersey dairy cows being divergent in residual feed intake and fed at 2 forage-to-concentrate ratios. J. Dairy Sci..

[68] Baldwin R.L., Wood W.A., Emery R.S. (1963). Conversion of Glucose-C14 To Propionate by the Rumen Microbiota. J. Bacteriol..

[69] Janssen P.H. (2010). Influence of hydrogen on rumen methane formation and fermentation balances through microbial growth kinetics and fermentation thermodynamics. Anim. Feed Sci. Technol..

[70] Li F., Li C., Chen Y., Liu J., Zhang C., Irving B., Fitzsimmons C., Plastow G., Guan L.L. (2019). Host genetics influence the rumen microbiota and heritable rumen microbial features associate with feed efficiency in cattle. Microbiome.

[71] Gaughan J.B., Holt S., Hahn G.L., Mader T.L., Eigenberg R. (2000). Respiration rate: Is it a good measure of heat stress in cattle?. Asian Australas. J. Anim. Sci..

[72] Dikmen S., Hansen P.J. (2009). Is the temperature-humidity index the best indicator of heat stress in lactating dairy cows in a subtropical environment?. J. Dairy Sci..

[73] Ammer S., Lambertz C., Gauly M. (2016). Comparison of different measuring methods for body temperature in lactating cows under different climatic conditions. J. Dairy Res..

[74] Jami E., Mizrahi I. (2012). Composition and similarity of bovine rumen microbiota across individual animals. PLoS ONE.

[75] Petri R.M., Schwaiger T., Penner G.B., Beauchemin K.A., Forster R.J., McKinnon J.J., McAllister T.A. (2013). Characterization of the core rumen microbiome in cattle during transition from forage to concentrate as well as during and after an acidotic challenge. PLoS ONE.

[76] Kim M., Yu Z. (2014). Variations in 16S rRNA-based microbiome profiling between pyrosequencing runs and between pyrosequencing facilities. J. Microbiol..

[77] Bharanidharan R., Arokiyaraj S., Bae Kim E., Hyun Lee C., Won Woo Y., Na Y., Kim D., Hoon Kim K. (2018). Ruminal methane emissions, metabolic, and microbial profile of Holstein steers fed forage and concentrate, separately or as a total mixed ration. PLoS ONE.

[78] Difford G.F., Plichta D.R., Løvendahl P., Lassen J., Noel S.J., Højberg O., Wright A.D.G., Zhu Z., Kristensen L., Nielsen H.B. (2018). Host genetics and the rumen microbiome jointly associate with methane emissions in dairy cows. PLoS Genet..

[79] Xue M., Sun H., Wu X., Guan L.L., Liu J. (2018). Assessment of rumen microbiota from a large dairy cattle cohort reveals the pan and core bacteriomes contributing to varied phenotypes. Appl. Environ. Microbiol..

[80] Kim M.S., Roh S.W., Nam Y.-D., Yoon J.H., Bae J.W. (2009). *Carnobacterium jeotgali* sp. nov., isolated from a Korean traditional fermented food. Int. J. Syst. Evol. Microbiol..

[81] Stewart C.S., Flint H.J., Bryant M.P. (1997). The rumen bacteria. The Rumen Microbial Ecosystem.

[82] Dassa B., Borovok I., Ruimy-Israeli V., Lamed R., Flint H.J., Duncan S.H., Henrissat B., Coutinho P., Morrison M., Mosoni P. (2014). Rumen cellulosomics: Divergent fiber-degrading strategies revealed by comparative genome-wide analysis of six ruminococcal strains. PLoS ONE.

[83] Baek Y.C., Choi H., Jeong J., Lee S.D., Kim M.J., Lee S., Ji S.Y., Kim M. (2020). The impact of short-term acute heat stress on the rumen microbiome of Hanwoo steers. J. Anim. Sci. Technol..

[84] Zhao S., Min L., Zheng N., Wang J. (2019). Effect of heat stress on bacterial composition and metabolism in the rumen of lactating dairy cows. Animals.

[85] Gonzalez-Recio O., Zubiria I., García-Rodríguez A., Hurtado A., Atxaerandio R. (2018). Short communication: Signs of host genetic regulation in the microbiome composition in 2 dairy breeds: Holstein and Brown Swiss. J. Dairy Sci..

[86] Bainbridge M.L., Cersosimo L.M., Wright A.D.G., Kraft J. (2016). Rumen bacterial communities shift across a lactation in Holstein, Jersey and Holstein × Jersey dairy cows and correlate to rumen function, bacterial fatty acid composition and production parameters. FEMS Microbiol. Ecol..

[87] Li R.W., Wu S., Baldwin R.L., Li W., Li C. (2012). Perturbation dynamics of the rumen microbiota in response to exogenous butyrate. PLoS ONE.

[88] Lee G.H., Rhee M.S., Chang D.H., Lee J., Kim S., Yoon M.H., Kim B.C. (2013). *Oscillibacter ruminantium* sp. nov., isolated from the rumen of Korean native cattle. Int. J. Syst. Evol. Microbiol..

[89] Hespell R.B. (1992). The Genera Succinivibrio and Succinimonas. The Prokaryotes.

[90] O’Herrin S.M., Kenealy W.R. (1993). Glucose and carbon dioxide metabolism by Succinivibrio dextrinosolvens. Appl. Environ. Microbiol..

