# Supplementary material for: Seasonal Influence on Rumen Microbiota, Rumen Fermentation, and Enteric Methane Emissions of Holstein and Jersey Steers under the Same Total Mixed Ration
Source: Animals (Basel). 2021 Apr 20;11(4):1184. doi: 10.3390/ani11041184 (PMC8074768; doi:10.3390/ani11041184)
Supplement: Supplementary file 1 [file animals-11-01184-s001.zip › animals-1154204-Table S1.pdf]

Table S1. Richness and diversity of rumen microbiome of Holstein and Jersey steers at different seasons.

Parameters	Breed	Season				SEM	Mixed p value	
		Winter	Spring	Summer	Overall		Season	Breed
OTUs	Hol	741.92	912.58	618.25	757.58	46.443	<0.01	0.40
	Jer	743.33	975.67	663.42	797.03	51.422		
	Total	746.96^{b}	944.13^{a}	640.83^{b}	-	48.649		
Chao1	Hol	854.09	1064.00	798.35	905.48	52.821	<0.01	0.55
	Jer	849.84	1135.61	834.77	944.19	54.175		
	Total	858.15^{b}	1099.81^{a}	816.56^{b}	-	53.106		
Shannon	Hol	5.63	6.46	6.77	6.29	0.198	0.01	0.85
	Jer	5.76	6.21	6.63	6.23	0.314		
	Total	5.74^{b}	6.33^{a}	6.70^{a}	-	0.261		
Inverse Simpson	Hol	0.92	0.96	0.96	0.95	0.010	0.02	0.68
	Jer	0.91	0.93	0.96	0.93	0.020		
	Total	0.92^{b}	0.94^{ab}	0.96^{a}	-	0.015		

OTUs, operational taxonomic units; SEM, standard error of the mean; Hol, Holstein steer; Jer, Jersey steer. $^{a, b, c}$ in the same row indicate the significant differences ($p < 0.05$) of data among three different seasons regardless of breed.
