# Supplementary material for: Seasonal Influence on Rumen Microbiota, Rumen Fermentation, and Enteric Methane Emissions of Holstein and Jersey Steers under the Same Total Mixed Ration
Source: Animals (Basel). 2021 Apr 20;11(4):1184. doi: 10.3390/ani11041184 (PMC8074768; doi:10.3390/ani11041184)
Supplement: Supplementary file 1 [file animals-11-01184-s001.zip › animals-1154204-Table S2.pdf]

Table S2. Major bacterial phyla identified in the rumen of the Holstein and the Jersey steers at different seasons.

**Bacterial Phyla**	Breed	Season				SEM	Mixed *p* value	
		Winter	Spring	Summer	Overall		Season	Breed
Bacteroidetes	Hol	57.91	68.89	58.98	61.93	2.994	<0.01	0.49
	Jer	50.96	70.08	67.31	62.78	2.416		
	Total	54.43[b]	69.48[a]	63.15[a]	-	2.705		
Firmicutes	Hol	35.81	17.84	28.45	27.37	2.650	<0.01	0.59
	Jer	43.53	21.30	26.14	30.32	2.913		
	Total	39.67[a]	19.57[c]	27.30[b]	-	2.782		
Proteobacteria	Hol	3.19	10.03	8.81	7.34	2.541	0.07	<0.01
	Jer	1.85	3.65	1.48	2.32	0.673		
	Total	2.52	6.84	5.14	-	1.607		
Tenericutes	Hol	1.26	0.82	0.48	0.86	0.206	0.04	0.83
	Jer	1.31	1.06	0.53	0.97	0.298		
	Total	1.29[a]	0.94[ab]	0.51[b]	-	0.252		
Spirochaetes	Hol	0.76	0.79	1.38	0.97	0.270	0.046	0.18
	Jer	0.52	2.83	3.31	2.22	0.798		
	Total	0.64[b]	1.81[ab]	2.34[a]	-	0.534		
Candidatus Melainabacteria	Hol	0.39	0.37	1.07	0.61	0.149	0.04	0.95
	Jer	0.62	0.37	0.48	0.49	0.096		
	Total	0.50[ab]	0.37[b]	0.77[a]	-	0.122		
Fibrobacteres	Hol	0.28	1.09	0.58	0.65	0.232	<0.01	0.07
	Jer	0.07	0.43	0.40	0.30	0.081		
	Total	0.17[b]	0.76[a]	0.49[ab]	-	0.157		

SEM, standard error of the mean; Hol, Holstein steer; Jer, Jersey steer. [a, b, c] in the same row indicate the significant differences ($p < 0.05$) of data among three different seasons regardless of breed.
