# Supplementary material for: Seasonal Influence on Rumen Microbiota, Rumen Fermentation, and Enteric Methane Emissions of Holstein and Jersey Steers under the Same Total Mixed Ration
Source: Animals (Basel). 2021 Apr 20;11(4):1184. doi: 10.3390/ani11041184 (PMC8074768; doi:10.3390/ani11041184)
Supplement: Supplementary file 1 [file animals-11-01184-s001.zip › animals-1154204-Table S3.pdf]

Table S3. Major genera of bacteria had relative abundance ≥1% at least in one breed at one season.

Phylum	Genera	Breed	Season				SEM	Mixed P value	
			Winter	Spring	Summer	Overall		Season	Breed
Bacteroidetes	*Prevotella*	Hol	45.45	46.85	35.60	42.63	3.036	0.03	0.57
		Jer	33.92	47.11	37.34	39.45	3.666		
		Total	39.68[ab]	46.98[a]	36.47[b]	-	3.351		
	Paraprevotella	Hol	4.48	4.59	2.72	3.39	0.723	0.049	0.24
		Jer	4.19[a]	3.89[a]	2.24[b]	3.44	0.889		
		Total	4.33	4.24	2.48	-	0.806		
	Paludibacter	Hol	1.82	4.07	8.66	4.85	1.409	<0.01	0.57
		Jer	1.48	3.00	9.74	4.74	1.234		
		Total	1.65[b]	3.54[b]	9.20[a]	-	1.321		
	Galbibacter	Hol	1.21	0.66	1.40	1.09	0.207	0.10	0.87
		Jer	1.10	0.98	1.32	1.13	0.260		
		Total	1.16	0.82	1.36	-	0.233		
	Bacteroides	Hol	1.08	2.67	0.81	1.52	0.268	<0.01	0.22
		Jer	0.93	2.09	0.65	1.22	0.413		
		Total	1.00[b]	2.38[a]	0.73[b]	-	0.341		
	Lentimicrobium	Hol	0.85	1.87	0.24	0.98	0.483	0.01	0.73
		Jer	0.79	0.40	0.33	0.51	0.090		
		Total	0.82[a]	1.14[a]	0.28[b]	-	0.287		
	Capnocytophaga	Hol	0.63	0.43	3.55	1.53	0.460	0.02	0.03
		Jer	0.96	4.35	3.41	2.91	0.937		
		Total	0.80[b]	2.39[ab]	3.48[a]	-	0.699		
	Muribaculum	Hol	0.31	0.80	0.18	0.43	0.189	0.19	0.03
		Jer	0.66	0.70	1.04	0.80	0.344		
		Total	0.48	0.75	0.61	-	0.267		
	Barnesiella	Hol	0.27	1.41	0.50	0.73	0.267	0.54	<0.01
		Jer	3.24	2.15	2.38	2.59	0.986		
		Total	1.75	1.78	1.44	-	0.626		
	Sphingobacterium	Hol	0.14	0.11	1.22	0.49	0.315	0.01	0.95
		Jer	0.43	0.11	3.05	1.20	0.846		
		Total	0.29[b]	0.11[b]	2.14[a]	-	0.580		
	Marseilla	Hol	0.14	2.20	1.03	1.12	0.499	<0.01	0.15
		Jer	0.88	1.30	1.85	1.34	0.408		
		Total	0.51[b]	1.75[a]	1.44[ab]	-	0.454		
	Parabacteroides	Hol	0.07	0.39	1.04	0.50	0.172	<0.01	0.43
		Jer	0.25	1.32	0.54	0.70	0.271		
		Total	0.16[b]	0.86[a]	0.79[a]	-	0.221		
	Olivibacter	Hol	0.00	0.00	0.00	0.00	0.001	0.02	0.13
		Jer	0.00	0.73	1.83	0.85	0.693		
		Total	0.00[b]	0.36[ab]	0.92[a]	-	0.347		
Candidatus Melainabacteria	*Vampirovibrio*	Hol	0.39	0.37	1.07	0.61	0.149	0.04	0.90
		Jer	0.59	0.37	0.48	0.48	0.100		
		Total	0.49[ab]	0.37[b]	0.77[a]	-	0.124		
Fibrobacteres	*Fibrobacter*	Hol	0.28	1.09	0.58	0.65	0.232	<0.01	0.08
		Jer	0.08	0.43	0.40	0.30	0.084		
		Total	0.18[b]	0.76[a]	0.49[ab]	-	0.158		
Firmicutes	*Carnobacterium*	Hol	11.83	0.00	0.00	3.94	0.554	<0.01	0.47

		Jer	7.94^{a}	0.00^{b}	0.00^{b}	2.65	1.219		
		Total	9.89	0.00	0.00	-	0.887		
		Hol	5.61	2.37	3.68	3.89	0.855		
	Ruminococcus	Jer	9.33	2.07	3.06	4.82	1.093	<0.01	0.83
		Total	7.47^{a}	2.22^{b}	3.37^{b}	-	0.974		
		Hol	2.43	0.55	1.27	1.42	0.250		
	Intestinimonas	Jer	2.91	0.69	1.89	1.83	0.328	<0.01	0.30
		Total	2.67^{a}	0.62^{c}	1.58^{b}	-	0.289		
		Hol	1.30	2.80	5.83	3.31	1.036		
	Succiniclasticum	Jer	1.30	2.70	2.50	2.17	0.643	0.01	0.51
		Total	1.30^{b}	2.75^{ab}	4.17^{a}	-	0.839		
		Hol	1.12	1.31	0.61	1.01	0.193		
	Ethanoligenens	Jer	2.37	1.43	1.01	1.60	0.672	0.03	0.41
		Total	1.74^{a}	1.37^{a}	0.81^{b}	-	0.432		
		Hol	1.07	0.18	0.25	0.50	0.230		
	Lactobacillus	Jer	0.18	0.16	0.08	0.14	0.059	0.55	0.05
		Total	0.63	0.17	0.17	-	0.144		
		Hol	0.95	0.61	1.46	1.01	0.179		
	Flintibacter	Jer	2.33	1.10	1.98	1.80	0.341	0.01	0.01
		Total	1.64^{a}	0.86^{b}	1.72^{a}	-	0.260		
		Hol	0.84	0.52	0.61	0.66	0.134		
	Christensenella	Jer	1.69	0.49	0.96	1.04	0.249	0.01	0.16
		Total	1.26^{a}	0.50^{b}	0.78^{ab}	-	0.191		
		Hol	0.84	0.51	0.61	0.65	0.101		
	UCG_Ruminococcaceae	Jer	2.37	0.60	1.16	1.38	0.378	0.01	0.03
		Total	1.60^{a}	0.55^{b}	0.88^{ab}	-	0.239		
		Hol	0.63	0.67	1.87	1.06	0.240		
	Anaerobacterium	Jer	0.64	0.78	1.45	0.96	0.253	0.01	0.80
		Total	0.64^{b}	0.73^{b}	1.66^{a}	-	0.246		
		Hol	0.30	0.58	0.80	0.56	0.097		
	Enterocloster	Jer	0.45	1.53	0.93	0.97	0.139	<0.01	0.02
		Total	0.37^{b}	1.06^{a}	0.87^{a}	-	0.118		
		Hol	0.20	0.62	1.47	0.76	0.142		
	Vallitalea	Jer	0.34	0.53	1.04	0.63	0.144	<0.01	0.75
		Total	0.27^{b}	0.57^{b}	1.26^{a}	-	0.143		
		Hol	0.17	0.19	0.76	0.37	0.093		
	Oscillibacter	Jer	0.75	1.11	1.08	0.98	0.317	0.01	<0.01
		Total	0.46^{b}	0.65^{ab}	0.92^{a}	-	0.205		
Proteobacteria		Hol	2.21	0.00	0.00	0.74	0.614		
	Pseudomonas	Jer	0.18	0.00	0.00	0.06	0.046	<0.01	0.36
		Total	1.19^{a}	0.00^{b}	0.00^{b}	-	0.330		
		Hol	0.36	2.87	0.86	1.36	0.257		
	Succinivibrio	Jer	0.23	0.98	0.67	0.62	0.389	0.01	0.01
		Total	0.29^{b}	1.92^{a}	0.77^{b}	-	0.323		
		Hol	0.29	5.93	7.41	4.54	1.772		
	Gilliamella	Jer	0.23	2.07	0.57	0.96	0.451	<0.01	0.02
		Total	0.26^{b}	4.00^{a}	3.99^{a}	-	1.112		
		Hol	0.75	0.75	1.36	0.95	0.270		
Spirochaetes	*Treponema*	Jer	0.47	2.76	3.27	2.17	0.795	0.05	0.22
		Total	0.61^{b}	1.76^{ab}	2.31^{a}	-	0.532		
Tenericutes	*Anaeroplasma*	Hol	0.83	0.45	0.38	0.55	0.136	0.17	0.73

Jer	1.04	0.46	0.42	0.64	0.249
Total	0.93	0.45	0.40	-	0.192

SEM, standard error of the mean; Hol, Holstein steer; Jer, Jersey steer. [a, b, c] in the same row indicate the significant differences ($p < 0.05$) of data among three different seasons regardless of breed.
